# Comparing Torque Transmission of Different Bracket Systems in Combination with Various Archwires Considering Play in the Bracket Slot: An In Vitro Study

**DOI:** 10.3390/ma17030684

**Published:** 2024-01-31

**Authors:** Andrea Wichelhaus, Simon Guggenbühl, Linus Hötzel, Corinna L. Seidel, Hisham Sabbagh, Lea Hoffmann

**Affiliations:** 1 Department of Orthodontics and Dentofacial Orthopedics, University Hospital, LMU Munich, Goethestrasse 70, 80366 Munich, Germany; kfo.sekretariat@med.uni-muenchen.de (A.W.); linus.hoetzel@med.uni-muenchen.de (L.H.); corinna.seidel@med.uni-muenchen.de (C.L.S.); hisham.sabbagh@med.uni-muenchen.de (H.S.); 2 Orthodontia Private Practice, Engelbergstrasse 28a, 6370 Stans, Switzerland; simon.guggenbuehl@gmail.com

**Keywords:** biomechanics, biomechanical phenomena, orthodontics, bracket slot, play, transmission, moment, torque, force

## Abstract

This study aims to examine the play between various archwires and bracket systems, exploring potential variations in angle values for specific torque and torque values for a given angle along different bracket systems. Therefore, seven brackets systems were evaluated in conjunction with different stainless steel archwires of varying dimensions (0.016″ × 0.022″, 0.018″ × 0.025″, and 0.019″ × 0.025″). Biomechanical behavior during torque development and transmission was assessed using a six-component force/torque sensor. Torque angles (5–45°) were specified with subsequent torque measurement, and the sequence was reversed by setting the torque (5–30 Nmm) and measuring the angle. A reference measurement with 0 Nmm torque served to evaluate bracket slot play. Bracket play (0 Nmm) during palatal load ranged between 20.06° and 32.50° for 0.016″ × 0.022″ wire, 12.83° and 21.11° for 0.018″ × 0.025″ wire, and 8.39° and 18.73° for 0.019″ × 0.025″ wire. The BioQuick^®^ bracket exhibited the highest play, while Wave SL^®^ and Damon^®^ Q brackets demonstrated the lowest play (*p* < 0.001). Significant differences (*p* < 0.001) between the brackets were observed in the torque angles required to achieve torques of 5–20 Nmm. In summary, each bracket system has a different torque transmission, which is of great clinical importance in order to achieve correct torque transmission and avoid complications such as root resorption.

## 1. Introduction

The straight-wire technique is the most commonly used in orthodontic therapy today. This method was developed in 1972 by Andrews [1], whereby all the necessary information for the alignment of a tooth in three dimensions was implemented in the bracket (bracket prescription). Various modifications to the straight-wire technique, including those proposed by Burstone [2] and Rickets [3], Roth [4], and Bennett and Mc Laughlin [5], aimed to enhance the original straight-wire system, by integrating torque, angulation, and offset in the brackets through different variations. Despite efforts, studies have demonstrated that torqute transmission does not sufficiently alter the axial position of teeth [6]. This limitation is mainly attributed to the play between the bracket slot and the archwire in use [7]. This play occurs due to the difference between the archwire and bracket dimension [8], leading to a deviation in torque transmission as the wire must rotate by a certain amount to get in contact with the wall of the slot for force transmission to occur. Thus, an additional torque must be applied for the correct axis position [1,9].

The designation of a “bent torque” refers to the twisting of the front part of the archwire in relation to the untwisted side segments of the wire. For example, with a 25° bent torque, the front part of the archwire is twisted by 25° in comparison to the rest of the archwire. Due to the pre-bent wire coupled with the prescription integrated into the bracket, a force reaction occurs between the archwire and bracket, because the archwire is inserted into the brackets against its pre-bend. This force results in a torque, which is decisive for the orthodontic movement of the teeth. In clinical practice, the torque bent into the archwires often lies between 20° and 30°, resulting in torques between 5 Nmm and 20 Nmm on the teeth [2]. The mean torsional play to achieve 20 Nmm was reported to be 18.5° for 0.016″ × 0.022″ wire [10] and 26.8° for 0.016″ × 0.016″ wire [11]. Another study showed a play of 4.5° for 0.020″ × 0.025″ wire and 11.7° for 0.019″ × 0.025″ wire, whereas a smaller slot leads to less play [12]. Furthermore, it was observed that the real play of the wire is 0.98–17.38° greater than the ideal [13].

However, torque transmission also varies depending on the type of bracket used. A distinction can be drawn between conventional twin brackets and self-ligating brackets [14]. In the case of conventional twin brackets, the connection between the wire and bracket is created by attached ligatures. Currently, two types of self-ligated systems are available on the market: active self-ligating and passive self-ligating systems [7]. Active systems have a clip-type closure mechanism that actively applies pressure to secure the archwire within the slot. In contrast, passive systems utilize a closing mechanism that essentially transforms the bracket into a tube. To date, there are inconsistent data on the influence of bracket type on torque transmission [7,15,16].

What follows is that different archwires can have different effects on the resulting torque. Beyond that, the various archwires available on the market have different characteristics in terms of slot geometry and material, which result in varying characteristics. These effects can influence the amount of transmitted torque and are therefore critical to the success of the treatment [12,13,17,18].

Another crucial point is the manufacturing tolerances in the production of orthodontic appliances, which leads to deviations from the standard geometry. Although characteristic features of the brackets and archwires are specified in inches, the tolerances lead to slightly different dimensions, for example, in the slot width or archwire geometry. Several studies have therefore shown that the geometries of brackets and wires have deviations contrary to the manufacturer’s specifications [10,11,13,19]. A study showed that an increase and decrease of the nominal dimensions can occur depending on the manufacturer and reported a range of −6.47% to +5.10% [13].

Moreover, when torque is applied to the bracket, plastic deformation of the bracket slot walls occurs. This involves beveling the edges and leads to an increase in the slot width in the upper slot area, which in turn leads to an increase in the play between the bracket and wire [11,19]. It has been reported that this has a significant impact on play and can occur in the same way with rectangular and square arches with a certain material dependency [13].

This study provides a comprehensive analysis of the significant differences in angle and torque values for all bracket–wire combinations investigated. The aim of this study is to investigate whether different brackets exhibit significant differences in play between the archwire and bracket slot. Furthermore, this study aims to determine if different brackets exhibit significant variations in angle values for a specific torque and, finally, whether there are significant differences in torque values for a certain angle among different brackets. Based on this study, clinicians can consider the unique characteristics of bracket–wire combinations to better assess torque transmission during treatment.

## 2. Materials and Methods

Seven different bracket systems and three archwires with different dimensions were tested and evaluated in terms of their biomechanical behavior during the development and transmission of torque (Table 1). The following bracket systems were considered within this study: two active self-ligating brackets made of stainless steel (SS) (Damon^®^ Q, Ormco, Orange, Ca; BioQuick^®^, Forestadent, Pforzheim, Germany), two active self-ligating brackets made of nickel–titanium (NiTi) (Wave SL^®^, Dentalline, Birkenfeld, Germany; 3M™ SmartClip™, 3M, Neuss, Germany), and two passive self-ligating brackets (In-Ovation^®^ C and In-Ovation^®^ R, Dentsply Sirona, York, PA, USA), as well as a SS twin bracket (Mini Sprint^®^, Forestadent, Pforzheim, Germany).

The bracket systems were investigated in combination with three stainless steel archwires with different dimensions: 0.016″ × 0.022″, 0.018″ × 0.025″, and 0.019″ × 0.025″ (Forestadent, Pforzheim, Germany).

**Figure 1:**
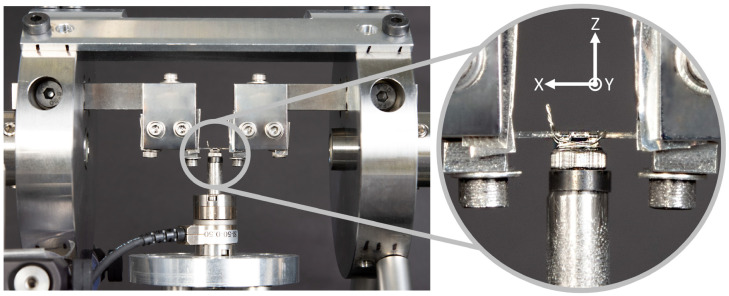
Experimental setup and coordinate system definition of the torque measurement device. The breakout focuses on the bracket with a ligated archwire on the sensor. The X-axis points along the bracket slot towards the left, the Z-axis points upwards as normal in the bracket slot, and the Y-axis is perpendicular to the previous axes.

The experimental setup and implemented coordinate system are shown in Figure 1. The coordinate system for the reference of the measurement data was defined so that the X-axis points along the bracket slot towards the left, the Z-axis points upwards as normal in the bracket slot, and the Y-axis is perpendicular to the previous axes. The same test setup and coordinate system was introduced in a previous publication by Wichelhaus [9]. During the experiment, the bracket was positioned on a sensor and the corresponding wire was inserted into the bracket. Torques and associated forces were measured in all three spatial directions using a six-component force/torque sensor (Nano17 SI-50-0.5, ATI Industrial Automation, Apex, NC, USA) with a force resolution of 0.0125 N and a torque resolution of 0.0625 Nmm. A stepper motor (QSH4218-41-10-035, Analog Devices Inc., Wilmington, MA, USA) with a corresponding motor driver was controlled via an Arduino^®^ Uno microcontroller. With the help of a mechanism and v-belt, the motor was used to rotate a horizontal element, into which the archwire was inserted. By moving the mechanics, the archwire could be horizontally adjusted to fit into the bracket slot. This made it possible to twist the archwire in the bracket slot around the X-axis with specified angles. The final control and collection of sensor data during the experiment was realized using LabVIEW (National Instruments Co., Austin, TX, USA).

The experiments were conducted within a temperature chamber set at T = 36 °C to replicate material behavior at body temperature. Each bracket–wire combination underwent *n* = 3 repetitions on independent samples in both the buccal and palatal directions. Each test run comprised three processes: the first involved specifying the angles with subsequent torque measurement, the second reversed the sequence by setting the torque and measuring the angles, and the third served as a reference measurement to evaluate bracket slot play by setting the torque to 0 Nmm.

In this study, three different wires of varying dimensions (0.016″ × 0.022″, 0.018″ × 0.025″, and 0.019″ × 0.025″) were assessed. Transmitting high forces and torques with smaller wires would inevitably lead to excessive twisting of the wire, which would no longer be clinically valid. Thus, the angles of the smallest archwire (0.016″ × 0.022″) were only measured from 0 Nmm to 15 Nmm, the angles of the 0.018″ × 0.025″ archwire were measured from 0 Nmm to 30 Nmm, and the angles of the thickest archwire (0.019″ × 0.025″) were measured from 0 Nmm to 40 Nmm. The opposite effect was evident for the twisted angles and the measured torque expression. 

Statistical analysis was performed using IBM SPSS Statistics 23 (IBM Corp., Armonk, NY, USA). Descriptive statistics including mean and standard deviation were calculated. A Shapiro–Wilk test was performed to check the data for normal distribution. Significant differences in metric data were analyzed using a Kruskal–Wallis test and additionally by the Dunn–Bonferroni post hoc test. A *p*-value < 0.05 was considered significant.

## 3. Results

**Figure 2:**
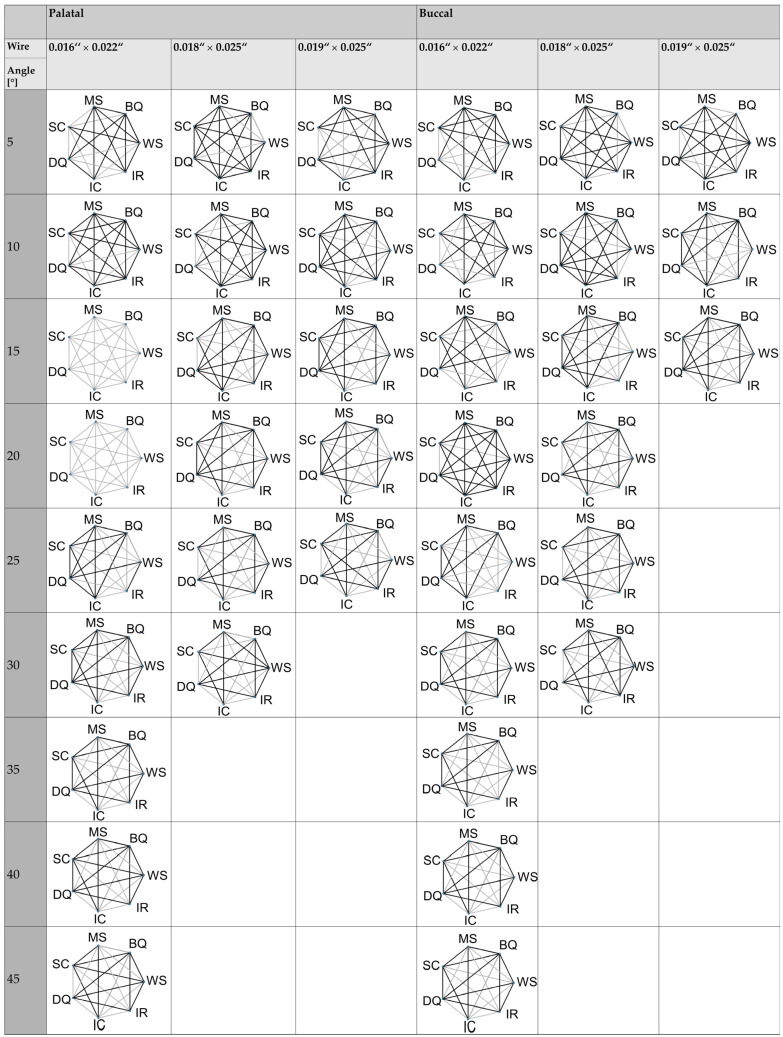
Differences between the brackets investigated at a given angle in degrees during a palatal and buccal load. DQ = Damon^®^ Q; BQ = BioQuick^®^; WS = Wave SL^®^; SC = SmartClip™; IC = In-Ovation^®^ C; IR = In-Ovation^®^ R; MS = Mini Sprint^®^. The black line indicates non-significant differences (*p* > 0.05) and the gray line indicates significant differences between the brackets.

The mean torque angles in degrees required to achieve various torques in Nmm using different brackets, along with the associated degrees of play, are presented in Table 2 (palatal load) and Table 3 (buccal load). Additionally, Figure 2 illustrates significant differences in measured torque among the investigated brackets with their respective wires and loads.

Depending on the bracket type, the bracket play (0 Nmm) ranged from 20.06° to 32.50° for the 0.016″ × 0.022″ wire dimension, 12.83° to 21.11° for the 0.018″ × 0.025″ wire dimension, and 8.39° to 18.73° for the 0.019″ × 0.025″ wire dimension during palatal loading (Table 2). During buccal loading, the bracket play (0 Nmm) ranged from 20.64° to 34.90° for the 0.016″ × 0.022″ wire dimension, 11.93° to 20.56° for the 0.018″ × 0.025″ wire dimension, and 6.63° to 18.54° for the 0.019″ × 0.025″ wire dimension (Table 3). The BioQuick^®^ bracket exhibited the highest play with all wires during palatal and buccal load (*p* < 0.001), while Wave SL^®^ and Damon^®^ Q showed the lowest play with all wires during palatal and buccal load (*p* < 0.001).

Significant differences (*p* < 0.001) in torque angles required to achieve different torque were observed among all investigated brackets during palatal or buccal load (Figure 2). When combined with a wire dimension of 0.016″ × 0.022″, the BioQuick^®^ bracket exhibited the highest required torque angles (36.72°, 41.09°, and 43.7°, respectively) to achieve torques of 5 Nmm, 10 Nmm, and 15 Nmm, while the Wave SL^®^ bracket showed the lowest torque angles (20.06°, 22.97°, 25.91°, and 28.75°, respectively) during buccal and palatal load. For the wire dimensions of 0.018″ × 0.025″ and 0.019″ × 0.025″, the BioQuick^®^ bracket also showed the highest required torque angles (21.11–31.07° and 18.73–31.2°, respectively) to achieve torques of 5 Nmm-40 Nmm. The Damon^®^ Q bracket exhibited the lowest required torque angles for the wire dimensions of 0.018″ × 0.025″ and 0.019″ × 0.025″ (14.56–23.13° and 10.13–21.05°, respectively) during buccal and palatal load.

**Figure 3:**
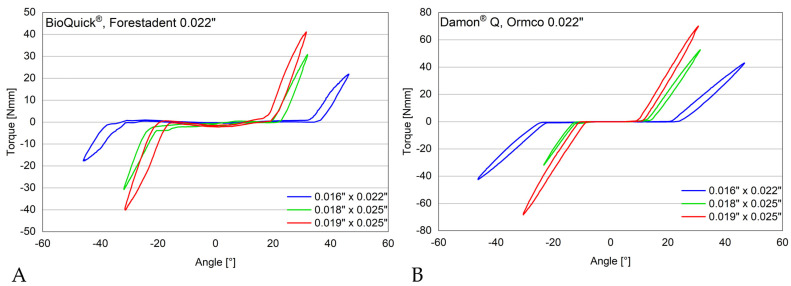
Example of torque measurements in Nmm. With increasing arch dimensions, exponentially high torque values occur with a small change in the torque angle. (**A**) BioQuick^®^ and (**B**) Damon^®^ Q bracket with a 0.022″ bracket slot in combination with 0.016″ × 0.022″, 0.018″ × 0.025″, and 0.019″ × 0.025″ wire.

**Figure 4:**
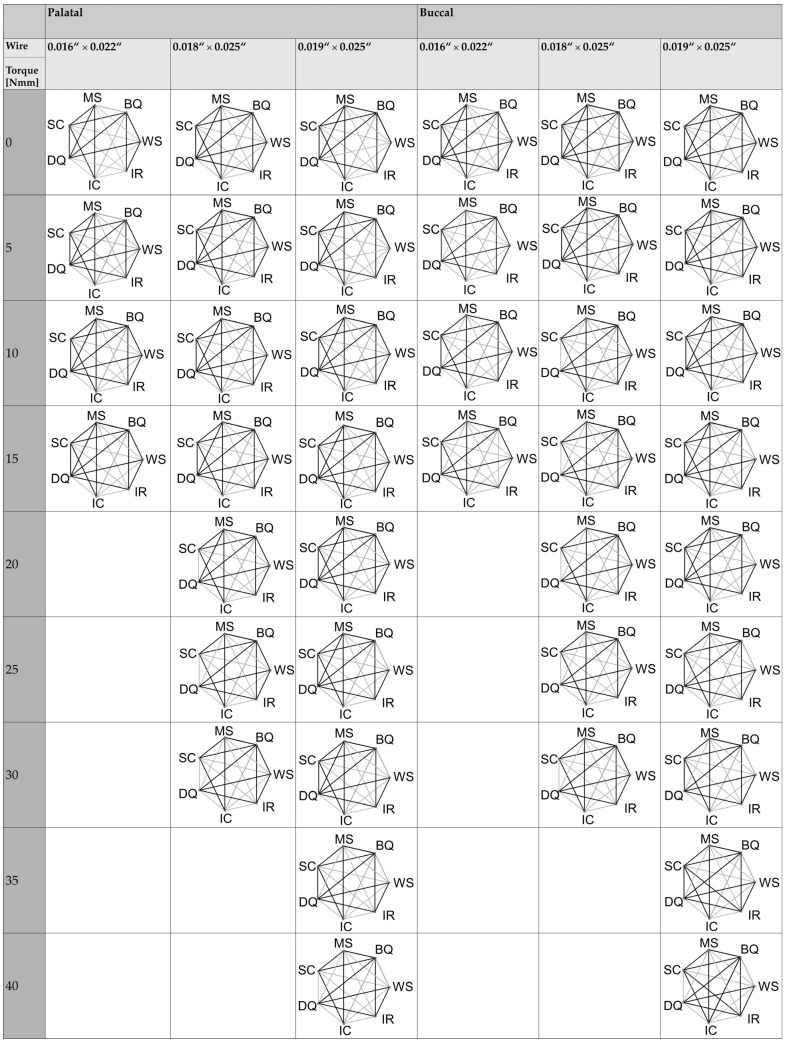
Differences in torque angles required to achieve different torque during palatal or buccal load between brackets investigated. Differences between the brackets investigated at a given angle in degrees during a palatal and buccal load. DQ = Damon^®^ Q; BQ = BioQuick^®^; WS = Wave SL^®^; SC = SmartClip™; IC = In-Ovation^®^ C; IR = In-Ovation^®^ R; MS = Mini Sprint^®^. The black line indicates non-significant differences (*p* > 0.05) and the gray line indicates significant differences between the brackets.

Figure 3 provides an example of the measured torques in Nmm required to achieve different angles (5–45°) using a BioQuick^®^ and Damon^®^ Q bracket with various wire dimensions. The assessed torque in Nmm to achieve different angles in degree using different brackets as well as the play in degree is shown in Table 4 (palatal load) and Table 5 (buccal load). Figure 4 depicts the significant differences for the measured angles between the brackets investigated with the different wires and different loads.

**Figure 5:**
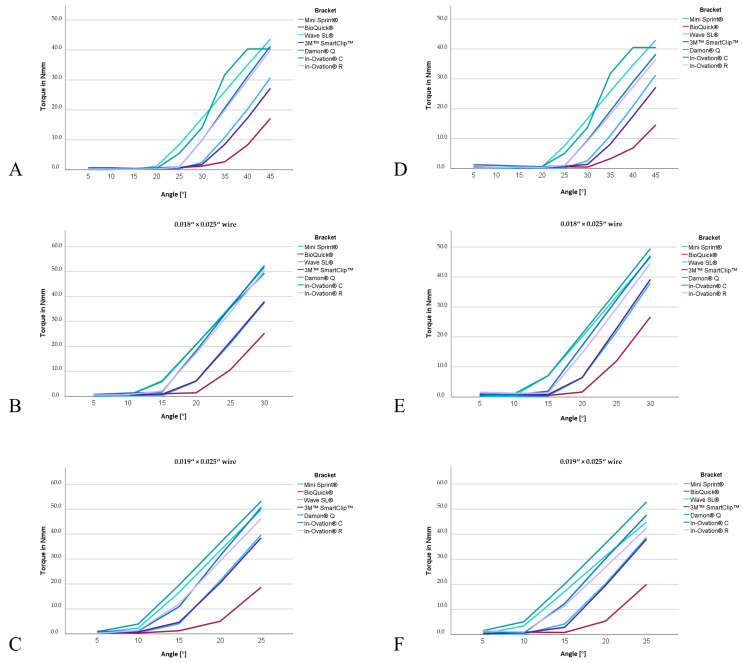
Measured torque in Nm with increasing angle in degree for all brackets examined in combination with (**A**,**D**): 0.016″ × 0.022″ wire; (**B**,**E**): 0.018″ × 0.025″ wire; (**C**,**F**): 0.019″ × 0.025″ wire. (**A**–**C**): palatal loading; (**D**–**F**): buccal loading.

With 0.016″ × 0.022″ wire, all investigated brackets showed no steady increase in torque between 5° and 15° (Figure 5). Above 20°, the torque in Nmm increased exponentially with increasing angle. It was only from 10° onwards that higher wire dimensions showed higher torques in all investigated brackets. For the 0.018″ × 0.025″ and 0.019″ × 0.025″ wire dimensions, a steady increase in torque with increasing angle can already be observed from 5° onwards.

With a wire dimension of 0.016″ × 0.022″, Mini Sprint^®^ achieved torques of 0.33 to 30.72 Nmm with a torque angle of 20–45°, while BioQuick^®^ achieved torques of 0.52–17.15 Nmm, Wave SL^®^ torques of 1.18–43.63 Nmm, SmartClip™ torques of 0.37–27.19 Nmm, Damon^®^ Q torques of 0.41–40.38 Nmm, In-Ovation^®^ C torques of 0.60–41.28 Nmm, and In-Ovation^®^ R torques of 0.29–39.97 Nmm. BioQuick^®^ reached the lowest torque of 17.15 Nmm at a maximal deflection of 45°, while the highest torques were reached by Damon^®^ Q (40.48 Nmm), In-Ovation^®^ C (41.28 Nmm), and Wave SL^®^ (43.63 Nmm) (*p* < 0.001). With a wire dimension of 0.018″ × 0.025″, BioQuick^®^ again achieved the lowest torque (25.20 Nmm), and In-Ovation^®^ R (50.35 Nmm), Wave SL^®^ (51.52 Nmm), and In-Ovation^®^ C (52.36 Nmm) achieved the highest torques (*p* < 0.001) using a maximal deflection of 30°. With a wire dimension of 0.019″ × 0.025″, BioQuick^®^ again achieved the lowest torques (18.68 Nmm), and Wave SL^®^ (49.80 Nmm), In-Ovation^®^ C (50.73 Nmm), and Damon^®^ Q (53.20 Nmm) achieved the highest torques (*p* < 0.001) with a maximal deflection of 25°.

**Figure 6:**
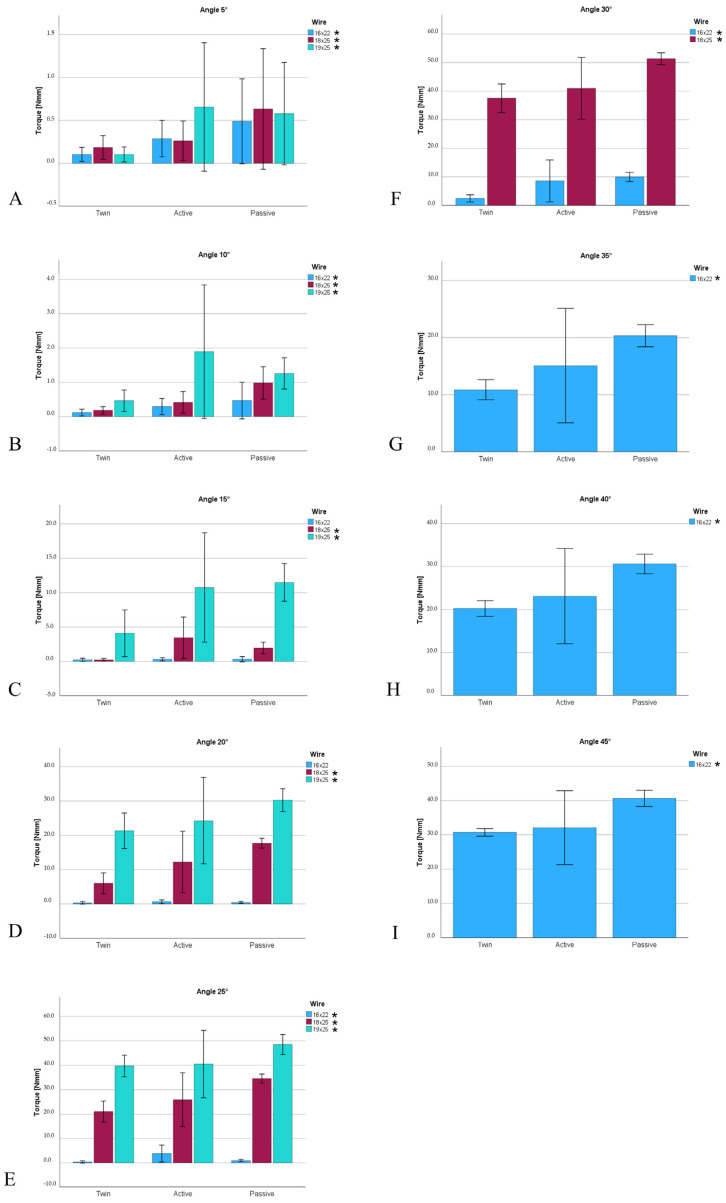
Comparison of the torques subdivided by the bracket types (Twin, Active, Passive). * Indicates significant differences between the bracket (*p* < 0.05) based on the Kruskal–Wallis test for independent samples. (**A**): 5° angle; (**B**): 10° angle; (**C**): 15° angle; (**D**): 20° angle; (**E**): 25° angle; (**F**): 30° angle; (**G**): 35° angle; (**H**): 40° angle; (**I**): 45° angle.

Figure 6 compares torques subdivided by bracket types (twin, active, passive). At angle values of 5° and 10°, the active brackets significantly showed the highest torques in Nmm, whereas at angle values of 15–40°, the passive brackets significantly exhibited the highest torques in Nmm.

## 4. Discussion

In this study, we observed significant differences in bracket play among all brackets investigated. The BioQuick^®^ bracket exhibited the highest play with all wires under both palatal and buccal loads (*p* < 0.001). Conversely, the Wave SL^®^ and Damon^®^ Q brackets demonstrated the lowest play with all wires during palatal and buccal loading (*p* < 0.001). These differences in bracket play have a substantial impact on the clinical outcomes, particularly in the context of active dynamic movements such as anterior tooth movement [20,21]. At low angles, torque is often not generated, depending on the bracket system used. The reason for this observation is that the play in the system has not been overcome, and in some systems, the wire rotates freely in the slot without making contact with the walls of the slot [22,23,24]. Therefore, this study underscores the necessity for different brackets to have distinct torque angles within different wires to prevent tipping during dynamic movements [21]. Thus, for example, a torque of at least 32.59° must be applied to the BioQuick^®^ bracket and a torque of 20.0° must be applied to the Wave SL^®^ using a 0.016″ × 0.022″ wire dimension in order to overcome the play and apply a torque. These findings are in line with Dalstra et al. [25], who postulated a play between 19.8° and 36.1° comparing conventional and self-ligating bracket systems.

Clinically effective torque has been suggested to range between 5 Nmm and 20 Nmm [2,26,27]. This study showed that using a wire dimension of 0.016″ × 0.022″, the BioQuick^®^ bracket required a torque angle of 36.72° to achieve the torque of 15 Nmm, while the bracket Wave SL^®^ required only 20.06°. Also, using a wire dimension of 0.018″ × 0.025″, the BioQuick^®^ bracket required the highest torque angle of 26.52° for 15 Nmm and 28.20° for 20 Nmm. In contrast, the Damon^®^ Q bracket only required a torque angle of 17.88° and 19.65° to achieve the same torques. It is therefore clinically important to know that a torque angle of 20° may have no effect at all on one bracket but may already have the maximum of the required Nmm using another bracket. These findings are in line with Morina et al. [28], who compared the torque expression of self-ligating brackets with conventional metallic, ceramic, and plastic brackets to show a wide range of required torque angles to achieve different torques in Nmm. 

However, having too much torque transmission carries inherent risks compared to having none at all. Generally, heightened magnitudes of applied forces and torques are linked to an augmented probability of unfavorable outcomes. The risk of root resorption during orthodontic treatment, despite its complex and multifactorial nature, is acknowledged, particularly in torquing movements where increased torque magnitudes are related to root resorptions. Casa et al. [29] observed that teeth subjected to higher torquing torques exhibited greater root resorption in terms of both width and depth, particularly concentrated in the apical third of the root. Furthermore, Bartley et al. [30] examined the degree of root resorption after 2.5° and 15° of buccal root torque over a four-week period. The study showed that root resorption was observed in both torque groups, concentrated in regions of compression. The torque bent into the archwires often lies between 20° and 30°. In the context of 0.018″ × 0.025″ wire, the present study revealed torques ranging from 20.6 Nmm to 51.5 Nmm when utilizing the Wave SL bracket. These values are nearly three times higher than expected, posing a substantial risk of root resorption [29,31,32,33,34].

To date, there are inconsistent findings in the literature on the influence of bracket types (twin, active, passive) on torque transmission. Brauchli et al. [7] investigated seven different self-ligated bracket systems and concluded that there were no significant differences between active and passive systems in torque expression. A systematic review showed that conventionally ligated systems exhibit higher torque expression than self-ligated systems, while active and passive systems show only marginal distinctions [27]. Badawi et al. [15] and Katsikogianna et al. [16] demonstrated that active brackets exhibited superior torque control compared to passive systems. The researchers concluded that active systems (In-Ovation and Speed) generated higher torquing torques than passive systems (Damon^®^ Q and SmartClip™) at clinically relevant torque angles (up to 35°). However, at higher (non-clinically relevant) angles, passive systems yielded greater torques. 

In this study, similar findings were observed. At lower angle values (5° or 10°), active brackets achieved higher torques. However, in contrast to Badawi et al. [15] and Katsikogianna et al. [16], higher torque transmission could be observed with the passive brackets from an angle of 15° onwards (Figure 4). However, what counters the influence of active and passive systems is that the active BioQuick^®^ bracket consistently showed the lowest torque values, indicating an influence due to the manufacturer rather than an influence by the bracket type (Figure 5).

The limitations of the current study lie in the transferability of the results from an in vitro analysis to a clinical in vivo situation. In the experimental setup of this study, the relevant wire was clamped in a single bracket. This approach differs from a clinical in vivo situation where the wire is held by multiple brackets. Consequently, there is a possibility that the generated torques in a clinical environment may vary.

Additionally, the present investigation was conducted under normal air conditions, whereas in a clinical application, the torque is applied in a moist environment. It has been documented that moisture and warmth in a simulated oral environment can have a significant impact on force transmission [35].

Furthermore, brackets in a clinical situation are rarely new when torque is applied, as they are usually introduced after the leveling phase. It is presumed that the effects of torque in a clinical application are lower due to the general usage of brackets compared to the results of this study. The fatigue of the flaps in self-ligating systems may also play a role over such an extended treatment period [36,37].

In addition, only steel wires were used in this study. Steel wires can already achieve greater torque transmission at a smaller angle compared to NiTi wires with the same magnitude. Archambault et al. [38] showed that, at an angle of 24 degrees, steel wires exhibit 1.5 to 2 times higher torque than TMA wires and 2.5 to 3 times higher torque than NiTi wires.

Therefore, further studies are recommended to, on the one hand, investigate torque transmission with multiple brackets in a humid environment and assess the influence of material fatigue and, on the other hand, analyze the impact of different wires on the torque transmission of the investigated brackets.

## 5. Conclusions

This study observed significant differences in bracket play, angle values, and torque values among all investigated brackets, which underscores the critical need for clinicians to consider the unique characteristics of each bracket–wire combination and precisely tailor torque angles in line with it. This customization is vital for avoiding undesirable side effects, particularly the risk of root resorptions, and enhancing the treatment efficacy to obtain the best clinical outcomes.

## Figures and Tables

**Table 1:** Brackets used throughout this study, showing trade name, manufacturer, material, and system of the brackets. All brackets were used in combination with 0.016″ × 0.022″, 0.018″ × 0.025″, and 0.019″ × 0.025″ wire.

Trade Name	Manufacturer	Material	System	Bracket Slot Width
Mini Sprint^®^	Forestadent	SS	Twin bracket	0.022″
BioQuick^®^	Forestadent	SS	SL passive	0.022″
Wave SL^®^	Dentalline	NiTi	SL passive	0.022″
3M™ SmartClip™	3M	NiTi	SL passive	0.022″
Damon^®^ Q	Ormco	SS	SL passive	0.022″
In-Ovation^®^ C	Dentsply Sirona	Ceramic	SL active	0.022″
In-Ovation^®^ R	Dentsply Sirona	SS	SL active	0.022″

(SS) = stainless steel; (NiTi) = nickel–titanium; (SL) = self-ligating.

**Table 2:** Mean torque angle and standard deviation in degrees to achieve torques of 5–40 Nmm with 0.016″ × 0.022″, 0.018″ × 0.025″, and 0.019″ × 0.025″ wire for different brackets during palatal loading. A *p*-value < 0.05 was considered significant (Kruskal–Wallis test). Post hoc analysis is pictured in Figure 2.

Wire	Bracket	Torque								
		0 Nmm	5 Nmm	10 Nmm	15 Nmm	20 Nmm	25 Nmm	30 Nmm	35 Nmm	40 Nmm
0.016″ × 0.022″	Mini Sprint^®^	28.77 (0.75)	31.65 (0.84)	34.52 (0.98)	37.31 (1.03)					
	BioQuick^®^	32.59 (4.83)	36.72 (2.6)	41.09 (1.42)	43.70 (1.31)					
	Wave SL^®^	20.06 (1.3)	22.97 (1.27)	25.91 (1.23)	28.75 (1.21)					
	SmartClip™	29.74 (0.39)	32.77 (0.35)	35.93 (0.55)	38.75 (0.65)					
	Damon^®^ Q	21.76 (0.41)	24.70 (0.42)	27.64 (0.45)	30.52 (0.48)					
	In-Ovation^®^ C	25.33 (0.87)	27.71 (0.89)	30.08 (0.95)	32.38 (1.0)					
	In-Ovation^®^ R	24.91 (0.36)	27.46 (0.48)	30.05 (0.67)	32.56 (0.77)					
	*p*-value	<0.001	<0.001	<0.001	<0.001					
0.018″ × 0.025″	Mini Sprint^®^	17.95 (0.98)	19.71 (1.09)	21.48 (1.2)	23.15 (1.32)	24.71 (1.41)	26.23 (1.44)	27.75 (1.51)		
	BioQuick^®^	21.11 (0.91)	22.92 (0.97)	24.83 (1.07)	26.52 (1.04)	28.20 (0.9)	29.86 (0.82)	31.07 (0.43)		
	Wave SL^®^	13.28 (1.5)	14.91 (1.41)	16.61 (1.36)	18.25 (1.39)	19.83 (1.38)	21.42 (1.34)	23.03 (1.35)		
	SmartClip™	17.99 (0.46)	19.61 (0.49)	21.33 (0.54)	22.90 (0.57)	24.45 (0.56)	25.99 (0.53)	27.52 (0.50)		
	Damon^®^ Q	12.83 (0.36)	14.56 (0.38)	16.35 (0.34)	18.07 (0.33)	19.74 (0.34)	21.44 (0.34)	23.13 (0.31)		
	In-Ovation^®^ C	14.85 (0.44)	16.28 (0.33)	17.69 (0.30)	19.13 (0.32)	20.51 (0.28)	21.93 (0.26)	23.36 (0.27)		
	In-Ovation^®^ R	14.59 (0.49)	16.19 (0.47)	17.81 (0.50)	19.36 (0.53)	20.9 (0.57)	22.41 (0.58)	23.92 (0.62)		
	*p*-value	<0.001	<0.001	<0.001	<0.001	<0.001	<0.001	<0.001		
0.019″ × 0.025″	Mini Sprint^®^	14.11 (1.13)	15.52 (1.19)	16.97 (1.26)	18.33 (1.34)	19.66 (1.38)	20.98 (1.27)	22.31 (1.37)	23.66 (1.33)	25.02 (1.27)
	BioQuick^®^	18.73 (1.23)	20.45 (1.56)	22.30 (1.85)	23.89 (1.95)	25.37 (1.91)	26.87 (1.79)	28.00 (1.03)	29.65 (0.75)	31.20 (0.72)
	Wave SL^®^	9.68 (1.12)	11.25 (1.01)	12.89 (0.93)	14.45 (0.87)	15.98 (0.82)	17.45 (0.77)	18.95 (0.71)	20.44 (0.66)	21.95 (0.6)
	SmartClip™	13.48 (0.35)	15.17 (0.4)	16.97 (0.54)	18.49 (0.67)	19.90 (0.74)	21.27 (0.77)	22.63 (0.77)	24.01 (0.75)	25.39 (0.70)
	Damon^®^ Q	8.39 (1.21)	10.13 (0.88)	12.08 (0.29)	13.61 (0.26)	15.13 (0.23)	16.63 (0.22)	18.09 (0.25)	19.58 (0.28)	21.05 (0.31)
	In-Ovation^®^ C	12.26 (0.79)	13.50 (0.80)	14.76 (0.81)	16.01 (0.81)	17.22 (0.85)	18.43 (0.84)	19.69 (0.9)	20.93 (0.92)	22.19 (0.93)
	In-Ovation^®^ R	11.37 (0.61)	12.86 (0.61)	14.38 (0.61)	15.91 (0.64)	17.35 (0.69)	18.81 (0.74)	20.29 (0.83)	21.73 (0.89)	23.18 (0.92)
	*p*-value	<0.001	<0.001	<0.001	<0.001	<0.001	<0.001	<0.001	<0.001	<0.001

**Table 3:** Mean torque angle and standard deviation in degrees to achieve torques of 5–40 Nmm with 0.016″ × 0.022″, 0.018″ × 0.025″, and 0.019″ × 0.025″ wire for different brackets during buccal loading. A *p*-value < 0.05 was considered significant (Kruskal–Wallis test). Post hoc analysis is pictured in Figure 2.

Wire	Bracket	Torque								
		0 Nmm	5 Nmm	10 Nmm	15 Nmm	20 Nmm	25 Nmm	30 Nmm	35 Nmm	40 Nmm
0.016″ × 0.022″	Mini Sprint^®^	28.87 (0.73)	31.64 (0.83)	34.48 (1.01)	37.08 (0.98)					
	BioQuick^®^	34.90 (3.56)	38.43 (1.99)	42.14 (1.47)	44.52 (0.78)					
	Wave SL^®^	20.64 (1.12)	23.48 (1.06)	26.32 (1.97)	29.09 (1.17)					
	SmartClip™	30.30 (0.63)	33.13 (0.49)	36.06 (0.44)	38.69 (0.51)					
	Damon^®^ Q	22.08 (0.64)	24.97 (0.60)	27.88 (0.62)	30.73 (0.61)					
	In-Ovation^®^ C	25.24 (0.63)	27.78 (0.72)	30.30 (0.81)	32.86 (0.90)					
	In-Ovation^®^ R	24.85 (0.46)	27.69 (0.55)	30.52 (0.70)	33.33 (0.79)					
	*p*-value	<0.001	<0.001	<0.001	<0.001					
0.018″ × 0.025″	Mini Sprint^®^	17.91 (0.91)	19.6 (1.03)	21.35 (1.18)	22.99 (1.25)	24.55 (1.29)	26.06 (1.32)	27.57 (1.36)		
	BioQuick^®^	20.51 (1.07)	22.39 (1.11)	24.36 (1.23)	26.04 (1.20)	27.68 (1.06)	29.37 (0.86)	31.01 (0.71)		
	Wave SL^®^	11.93 (1.25)	14.14 (1.16)	16.38 (1.31)	18.31 (1.44)	20.21 (1.75)	21.98 (1.81)	23.88 (1.96)		
	SmartClip™	17.92 (0.33)	19.51 (0.31)	21.14 (0.36)	22.69 (0.38)	24.20 (0.41)	25.70 (0.43)	27.19 (0.44)		
	Damon^®^ Q	12.39 (0.36)	14.19 (0.31)	16.05 (0.30)	17.88 (0.34)	19.65 (0.37)	21.41 (0.39)	23.15 (0.42)		
	In-Ovation^®^ C	14.48 (0.55)	16.10 (0.53)	17.73 (0.54)	19.31 (0.53)	21.02 (0.56)	22.62 (0.52)	24.25 (0.50)		
	In-Ovation^®^ R	15.04 (0.54)	16.74 (0.60)	18.45 (0.67)	20.16 (0.70)	21.85 (0.72)	23.51 (0.71)	25.16 (0.69)		
	*p*-value	<0.001	<0.001	<0.001	<0.001	<0.001	<0.001	<0.001		
0.019″ × 0.025″	Mini Sprint^®^	14.30 (1.7)	15.71 (1.75)	17.17 (1.80)	18.6 (1.91)	19.96 (1.92)	21.30 (1.91)	22.62 (1.88)	23.92 (1.84)	25.23 (1.71)
	BioQuick^®^	18.54 (1.16)	20.15 (1.38)	21.94 (1.54)	23.43 (1.63)	24.87 (1.59)	26.38 (1.47)	27.96 (1.26)	29.63 (1.01)	30.87 (1.15)
	Wave SL^®^	9.05 (1.25)	10.74 (1.09)	12.55 (0.97)	14.25 (0.91)	15.95 (0.90)	17.73 (0.85)	19.54 (0.91)	21.37 (0.96)	23.23 (1.04)
	SmartClip™	14.25 (0.36)	15.76 (0.33)	17.34 (0.38)	18.78 (0.47)	20.13 (0.56)	21.50 (0.58)	22.84 (0.57)	24.18 (0.56)	25.52 (0.52)
	Damon^®^ Q	6.63 (3.73)	8.92 (2.44)	11.79 (0.56)	13.40 (0.56)	14.98 (0.58)	16.52 (0.56)	18.05 (0.57)	19.59 (0.57)	21.12 (0.58)
	In-Ovation^®^ C	11.58 (0.56)	12.96 (0.56)	14.34 (0.58)	15.73 (0.59)	17.12 (0.60)	18.50 (0.59)	19.92 (0.60)	21.33 (0.58)	22.76 (0.56)
	In-Ovation^®^ R	11.25 (0.78)	12.87 (0.78)	14.51 (0.79)	16.14 (0.81)	17.77 (0.84)	19.40 (0.84)	20.99 (0.84)	22.60 (0.82)	24.20 (0.81)
	*p*-value	<0.001	<0.001	<0.001	<0.001	<0.001	<0.001	<0.001	<0.001	<0.001

**Table 4:** Mean torque and standard deviation in Nmm to achieve angles of 5–45° with 0.016″ × 0.022″, 0.018″ × 0.025″, and 0.019″ × 0.025″ wire for different brackets during palatal loading. A *p*-value < 0.05 was considered significant (Kruskal–Wallis test). Post hoc analysis is pictured in Figure 4.

Wire	Bracket	Angle								
		5°	10°	15°	20°	25°	30°	35°	40°	45°
0.016″ × 0.022″	Mini Sprint^®^	0.10 (0.08)	0.12 (0.10)	0.23 (0.23)	0.33 (0.37)	0.33 (0.49)	2.47 (1.27)	10.88 (1.75)	20.27 (1.84)	30.72 (1.11)
	BioQuick^®^	0.51 (0.19)	0.52 (0.24)	0.42 (0.25)	0.52 (0.19)	0.72 (0.14)	1.23 (1.06)	2.69 (2.11)	8.31 (2.37)	17.15 (1.90)
	Wave SL^®^	0.17 (0.04)	0.17 (0.05)	0.17 (0.11)	1.18 (0.82)	8.42 (2.17)	17.23 (2.21)	26.19 (2.14)	35.07 (1.96)	43.63 (1.68)
	SmartClip™	0.33 (0.23)	0.36 (0.27)	0.37 (0.31)	0.37 (0.37)	0.72 (0.38)	1.79 (0.62)	8.51 (0.91)	17.32 (1.11)	27.19 (0.83)
	Damon^®^ Q	0.14 (0.08)	0.13 (0.06)	0.24 (0.10)	0.41 (0.13)	5.51 (0.72)	14.08 (0.85)	22.98 (0.86)	31.79 (0.66)	40.38 (0.37)
	In-Ovation^®^ C	0.69 (0.65)	0.69 (0.69)	0.42 (0.51)	0.60 (0.33)	0.96 (0.47)	9.91 (1.99)	20.67 (2.16)	31.20 (2.35)	41.28 (2.55)
	In-Ovation^®^ R	0.29 (0.09)	0.25 (0.11)	0.20 (0.14)	0.29 (0.15)	0.91 (0.51)	10.00 (1.24)	19.95 (1.71)	30.07 (2.12)	39.97 (2.37)
	*p*-value	<0.001	<0.001	0.17	0.26	<0.001	<0.001	<0.001	<0.001	<0.001
0.018″ × 0.025″	Mini Sprint^®^	0.18 (0.14)	0.18 (0.11)	0.22 (0.20)	6.03 (3.02)	21.10 (4.28)	37.53 (5.00)			
	BioQuick^®^	0.34 (0.34)	0.60 (0.38)	1.04 (0.78)	1.43 (0.91)	10.58 (3.15)	25.20 (2.19)			
	Wave SL^®^	0.20 (0.14)	0.30 (0.27)	5.79 (3.01)	20.59 (4.26)	36.07 (4.14)	51.52 (4.12)			
	SmartClip™	0.42 (0.12)	0.46 (0.28)	0.82 (0.61)	6.16 (1.44)	21.79 (1.80)	37.93 (1.37)			
	Damon^®^ Q	0.09 (0.08)	0.29 (0.24)	6.13 (0.99)	20.73 (1.93)	35.36 (0.88)	49.33 (0.47)			
	In-Ovation^®^ C	0.76 (0.97)	1.28 (0.36)	1.76 (0.94)	18.15 (0.89)	35.54 (0.87)	52.36 (1.04)			
	In-Ovation^®^ R	0.50 (0.23)	0.68 (0.37)	2.15 (0.72)	17.25 (1.73)	33.69 (2.09)	50.35 (2.40)			
	*p*-value	<0.001	<0.001	<0.001	<0.001	<0.001	<0.001			
0.019″ × 0.025″	Mini Sprint^®^	0.10 (0.09)	0.46 (0.32)	4.10 (3.41)	21.33 (5.18)	39.70 (4.42)				
	BioQuick^®^	1.07 (0.27)	0.37 (0.22)	1.26 (0.70)	5.07 (3.23)	18.69 (5.56)				
	Wave SL^®^	0.51 (0.27)	2.31 (2.03)	16.68 (2.77)	33.49 (2.23)	49.80 (1.54)				
	SmartClip™	0.19 (0.11)	0.82 (0.75)	4.67 (1.00)	20.44 (2.70)	38.50 (2.57)				
	Damon^®^ Q	0.88 (1.31)	3.95 (1.64)	19.62 (0.08)	36.49 (0.95)	53.20 (1.40)				
	In-Ovation^®^ C	0.91 (0.66)	1.13 (0.48)	10.93 (3.20)	31.29 (3.57)	50.73 (3.50)				
	In-Ovation^®^ R	0.25 (0.29)	1.39 (0.41)	12.04 (2.15)	29.22 (2.87)	46.19 (3.32)				
	*p*-value	<0.001	<0.001	<0.001	<0.001	<0.001				

**Table 5:** Mean torque and standard deviation in Nmm to achieve angles of 5–45° with 0.016″ × 0.022″, 0.018″ × 0.025″, and 0.019″ × 0.025″ wire for different brackets during buccal loading. A *p*-value < 0.05 was considered significant (Kruskal–Wallis test). Post hoc analysis is pictured in Figure 4.

Wire	Bracket	Angle								
		5°	10°	15°	20°	25°	30°	35°	40°	45°
0.016″ × 0.022″	Mini Sprint^®^	0.12 (0.10)	0.10 (0.10)	0.11 (0.06)	0.15 (0.09)	0.22 (0.18)	2.62 (1.08)	10.99 (1.91)	20.95 (1.61)	31.31 (1.00)
	BioQuick^®^	0.49 (0.18)	0.37 (0.17)	0.18 (0.17)	0.30 (0.11)	0.62 (0.28)	0.51 (0.38)	3.28 (1.61)	6.90 (2.11)	14.62 (1.91)
	Wave SL^®^	0.15 (0.02)	0.12 (0.02)	0.09 (0.05)	0.49 (0.50)	7.67 (1.84)	16.72 (2.14)	25.79 (2.44)	34.65 (2.44)	42.97 (2.29)
	SmartClip™	0.25 (0.08)	0.26 (0.10)	0.29 (0.15)	0.40 (0.25)	0.77 (0.48)	1.40 (0.94)	8.14 (0.75)	17.55 (0.91)	27.19 (0.52)
	Damon^®^ Q	0.11 (0.05)	0.22 (0.16)	0.45 (0.13)	0.67 (0.10)	5.10 (0.94)	13.75 (1.12)	22.64 (1.13)	31.77 (1.06)	40.47 (0.83)
	In-Ovation^®^ C	1.28 (0.94)	1.08 (0.69)	0.82 (0.54)	0.65 (0.57)	0.94 (0.62)	9.44 (1.51)	19.20 (1.76)	29.00 (1.83)	38.22 (2.16)
	In-Ovation^®^ R	0.21 (0.12)	0.31 (0.11)	0.48 (0.07)	0.39 (0.23)	1.07 (0.55)	9.15 (1.20)	18.07 (1.52)	27.34 (1.77)	36.56 (2.05)
	*p*-value	<0.001	<0.001	<0.001	<0.001	<0.001	<0.001	<0.001	<0.001	<0.001
0.018″ × 0.025″	Mini Sprint^®^	0.20 (0.13)	0.23 (0.14)	0.32 (0.26)	6.45 (2.91)	21.63 (4.09)	38.02 (4.50)			
	BioQuick^®^	1.03 (0.62)	0.78 (0.66)	0.53 (0.83)	1.66 (1.79)	11.89 (3.37)	26.68 (2.19)			
	Wave SL^®^	0.23 (0.17)	0.33 (0.33)	7.13 (2.42)	20.06 (3.88)	33.40 (5.37)	46.57 (6.20)			
	SmartClip™	0.61 (0.16)	0.75 (0.37)	0.82 (0.65)	6.50 (0.99)	22.71 (1.42)	39.22 (1.78)			
	Damon^®^ Q	0.37 (0.34)	1.02 (0.32)	7.11 (0.81)	21.02 (1.07)	35.18 (1.19)	49.48 (1.10)			
	In-Ovation^®^ C	1.00 (0.99)	0.74 (0.57)	1.92 (1.26)	17.08 (1.65)	32.29 (1.44)	47.06 (1.45)			
	In-Ovation^®^ R	1.60 (0.28)	1.27 (0.33)	1.30 (1.12)	14.56 (2.07)	29.48 (2.12)	44.59 (2.09)			
	*p*-value	<0.001	<0.001	<0.001	<0.001	<0.001	<0.001			
0.019″ × 0.025″	Mini Sprint^®^	0.13 (0.07)	0.22 (0.11)	4.17 (3.09)	20.29 (6.70)	38.79 (6.69)				
	BioQuick^®^	1.11 (0.57)	0.94 (0.30)	0.80 (1.03)	5.35 (3.75)	19.98 (5.01)				
	Wave SL^®^	0.35 (0.29)	3.40 (2.42)	17.16 (2.52)	31.32 (2.26)	44.82 (2.83)				
	SmartClip™	0.26 (0.09)	0.69 (0.70)	2.84 (1.09)	19.57 (1.98)	38.00 (2.00)				
	Damon^®^ Q	1.50 (2.13)	5.08 (2.09)	20.13 (1.82)	36.34 (1.90)	52.85 (2.08)				
	In-Ovation^®^ C	1.23 (0.83)	0.54 (0.65)	12.33 (2.14)	30.29 (2.07)	47.64 (1.84)				
	In-Ovation^®^ R	1.41 (0.23)	1.05 (0.28)	11.52 (2.54)	26.89 (2.61)	42.48 (2.44)				
	*p*-value	<0.001	<0.001	<0.001	<0.001	<0.001				

## Data Availability

The raw data supporting the conclusions of this article will be made available by the authors on request.

## References

[1] Andrews L.F. (1972). The six keys to normal occlusion. Am. J. Orthod..

[2] Burstone C.J. (1966). The mechanics of the segmented arch techniques. Angle Orthod..

[3] Rickets R.M. (1979). Bioprogressive Therapy.

[4] Roth R.H. (1976). The maintenance system and occlusal dynamics. Dent. Clin. N. Am..

[5] Bennett J.C., McLaughlin R.P. (1990). Controlled space closure with a preadjusted appliance system. J. Clin. Orthod..

[6] Mittal M., Thiruvenkatachari B., Sandler P.J., Benson P.E. (2015). A three-dimensional comparison of torque achieved with a preadjusted edgewise appliance using a Roth or MBT prescription. Angle Orthod..

[7] Brauchli L.M., Steineck M., Wichelhaus A. (2012). Active and passive self-ligation: A myth? Part 1: Torque control. Angle Orthod..

[8] Arreghini A., Lombardo L., Mollica F., Siciliani G. (2014). Torque expression capacity of 0.018 and 0.022 bracket slots by changing archwire material and cross section. Prog. Orthod..

[9] Wichelhaus A. (2017). A new elastic slot system and V-wire mechanics. Angle Orthod..

[10] Meling T.R., Odegaard J., Meling E.O. (1997). On mechanical properties of square and rectangular stainless steel wires tested in torsion. Am. J. Orthod. Dentofac. Orthop..

[11] Meling T.R., Odegaard J. (1998). The effect of cross-sectional dimensional variations of square and rectangular chrome-cobalt archwires on torsion. Angle Orthod..

[12] Joch A., Pichelmayer M., Weiland F. (2010). Bracket slot and archwire dimensions: Manufacturing precision and third order clearance. J. Orthod..

[13] Lombardo L., Arreghini A., Bratti E., Mollica F., Spedicato G., Merlin M., Fortini A., Siciliani G. (2015). Comparative analysis of real and ideal wire-slot play in square and rectangular archwires. Angle Orthod..

[14] Turnbull N.R., Birnie D.J. (2007). Treatment efficiency of conventional vs self-ligating brackets: Effects of archwire size and material. Am. J. Orthod. Dentofac. Orthop..

[15] Badawi H.M., Toogood R.W., Carey J.P., Heo G., Major P.W. (2008). Torque expression of self-ligating brackets. Am. J. Orthod. Dentofac. Orthop..

[16] Katsikogianni E.N., Reimann S., Weber A., Karp J., Bourauel C. (2015). A comparative experimental investigation of torque capabilities induced by conventional and active, passive self-ligating brackets. Eur. J. Orthod..

[17] Papageorgiou S.N., Sifakakis I., Doulis I., Eliades T., Bourauel C. (2016). Torque efficiency of square and rectangular archwires into 0.018 and 0.022 in. conventional brackets. Prog. Orthod..

[18] Sifakakis I., Pandis N., Makou M., Eliades T., Katsaros C., Bourauel C. (2014). Torque efficiency of different archwires in 0.018- and 0.022-inch conventional brackets. Angle Orthod..

[19] Fischer-Brandies H., Orthuber W., Es-Souni M., Meyer S. (2000). Torque transmission between square wire and bracket as a function of measurement, form and hardness parameters. J. Orofac. Orthop..

[20] Tominaga J.Y., Chiang P.C., Ozaki H., Tanaka M., Koga Y., Bourauel C., Yoshida N. (2012). Effect of play between bracket and archwire on anterior tooth movement in sliding mechanics: A three-dimensional finite element study. J. Dent. Biomech..

[21] Tominaga J.Y., Ozaki H., Chiang P.C., Sumi M., Tanaka M., Koga Y., Bourauel C., Yoshida N. (2014). Effect of bracket slot and archwire dimensions on anterior tooth movement during space closure in sliding mechanics: A 3-dimensional finite element study. Am. J. Orthod. Dentofac. Orthop..

[22] Odegaard J., Meling T., Meling E. (1994). An evaluation of the torsional moments developed in orthodontic applications. An in vitro study. Am. J. Orthod. Dentofac. Orthop..

[23] Huang Y., Keilig L., Rahimi A., Reimann S., Eliades T., Jager A., Bourauel C. (2009). Numeric modeling of torque capabilities of self-ligating and conventional brackets. Am. J. Orthod. Dentofac. Orthop..

[24] Hirai M., Nakajima A., Kawai N., Tanaka E., Igarashi Y., Sakaguchi M., Sameshima G.T., Shimizu N. (2012). Measurements of the torque moment in various archwire-bracket-ligation combinations. Eur. J. Orthod..

[25] Dalstra M., Eriksen H., Bergamini C., Melsen B. (2015). Actual versus theoretical torsional play in conventional and self-ligating bracket systems. J. Orthod..

[26] Major T.W., Carey J.P., Nobes D.S., Heo G., Major P.W. (2011). Mechanical effects of third-order movement in self-ligated brackets by the measurement of torque expression. Am. J. Orthod. Dentofac. Orthop..

[27] Al-Thomali Y., Mohamed R.N., Basha S. (2017). Torque expression in self-ligating orthodontic brackets and conventionally ligated brackets: A systematic review. J. Clin. Exp. Dent..

[28] Morina E., Eliades T., Pandis N., Jager A., Bourauel C. (2008). Torque expression of self-ligating brackets compared with conventional metallic, ceramic, and plastic brackets. Eur. J. Orthod..

[29] Casa M.A., Faltin R.M., Faltin K., Sander F.G., Arana-Chavez V.E. (2001). Root resorptions in upper first premolars after application of continuous torque moment. Intra-individual study. J. Orofac. Orthop..

[30] Bartley N., Turk T., Colak C., Elekdag-Turk S., Jones A., Petocz P., Darendeliler M.A. (2011). Physical properties of root cementum: Part 17. Root resorption after the application of 2.5 degrees and 15 degrees of buccal root torque for 4 weeks: A microcomputed tomography study. Am. J. Orthod. Dentofac. Orthop..

[31] Wichelhaus A., Dulla M., Sabbagh H., Baumert U., Stocker T. (2021). Stainless steel and NiTi torque archwires and apical root resorption. J. Orofac. Orthop..

[32] Faltin R.M., Arana-Chavez V.E., Faltin K., Sander F.G., Wichelhaus A. (1998). Root resorptions in upper first premolars after application of continuous intrusive forces. Intra-individual study. J. Orofac. Orthop..

[33] Nakano T., Hotokezaka H., Hashimoto M., Sirisoontorn I., Arita K., Kurohama T., Darendeliler M.A., Yoshida N. (2014). Effects of different types of tooth movement and force magnitudes on the amount of tooth movement and root resorption in rats. Angle Orthod..

[34] Roscoe M.G., Meira J.B., Cattaneo P.M. (2015). Association of orthodontic force system and root resorption: A systematic review. Am. J. Orthod. Dentofac. Orthop..

[35] Taloumis L.J., Smith T.M., Hondrum S.O., Lorton L. (1997). Force decay and deformation of orthodontic elastomeric ligatures. Am. J. Orthod. Dentofac. Orthop..

[36] Major T.W., Carey J.P., Nobes D.S., Heo G., Major P.W. (2012). Deformation and warping of the bracket slot in select self-ligating orthodontic brackets due to an applied third order torque. J. Orthod..

[37] Major T.W., Carey J.P., Nobes D.S., Heo G., Melenka G.W., Major P.W. (2013). An investigation into the mechanical characteristics of select self-ligated brackets at a series of clinically relevant maximum torquing angles: Loading and unloading curves and bracket deformation. Eur. J. Orthod..

[38] Archambault A., Major T.W., Carey J.P., Heo G., Badawi H., Major P.W. (2010). A comparison of torque expression between stainless steel, titanium molybdenum alloy, and copper nickel titanium wires in metallic self-ligating brackets. Angle Orthod..

